# Systematic review and meta-analysis of experimental studies evaluating the organ protective effects of histone deacetylase inhibitors

**DOI:** 10.1016/j.trsl.2018.11.002

**Published:** 2019-03

**Authors:** Syabira I. Yusoff, Marius Roman, Florence Y. Lai, Bryony Eagle-Hemming, Gavin J. Murphy, Tracy Kumar, Marcin Wozniak

**Affiliations:** Department of Cardiovascular Sciences and NIHR Leicester Biomedical Research Unit in Cardiovascular Medicine, University of Leicester, Clinical Sciences Wing, Glenfield Hospital, Leicester, UK

## Abstract

The clinical efficacy of organ protection interventions are limited by the redundancy of cellular activation mechanisms. Interventions that target epigenetic mechanisms overcome this by eliciting genome wide changes in transcription and signaling. We aimed to review preclinical studies evaluating the organ protection effects of histone deacetylase inhibitors (HDACi) with a view to informing the design of early phase clinical trials. A systematic literature search was performed. Methodological quality was assessed against prespecified criteria. The primary outcome was mortality, with secondary outcomes assessing mechanisms. Prespecified analyses evaluated the effects of likely moderators on heterogeneity. The analysis included 101 experimental studies in rodents (n = 92) and swine (n = 9), exposed to diverse injuries, including: ischemia (n = 72), infection (n = 7), and trauma (n = 22). There were a total of 448 comparisons due to the evaluation of multiple independent interventions within single studies. Sodium valproate (VPA) was the most commonly evaluated HDACi (50 studies, 203 comparisons). All of the studies were judged to have significant methodological limitations. HDACi reduced mortality in experimental models of organ injury (risk ratio = 0.52, 95% confidence interval 0.40–0.68, p < 0.001) without heterogeneity. HDACi administration resulted in myocardial, brain and kidney protection across diverse species and injuries that was attributable to increases in prosurvival cell signaling, and reductions in inflammation and programmed cell death. Heterogeneity in the analyses of secondary outcomes was explained by differences in species, type of injury, HDACi class (Class I better), drug (trichostatin better), and time of administration (at least 6 hours prior to injury better). These findings highlight a potential novel application for HDACi in clinical settings characterized by acute organ injury.

## INTRODUCTION

Decades of research have yielded multiple negative clinical trials of organ protection interventions.^1^, ^2^^,^^3^ A major challenge in this field is to overcome the redundancy of the multiple pathways activated in response to injury using a single intervention.^2^ Interventions targeting epigenetic processes offer a possible solution. Modification of the regulation of gene expression through alterations in chromatin components other than the DNA sequence can regulate the expression of multiple gene pathways that determine stress responses, energy utilization, and cell survival.^4^ Multiple epigenetic mechanisms exist ranging from DNA methylation which elicits long-term changes in the genome to processes with greater plasticity such as histone acetylation and deacetylation. These processes are strongly influenced by adverse environmental stimuli and have evolved to modulate a genome wide response to stress. The ability to modify epigenetic processes raises the possibility of harnessing this genome wide response as an organ protection intervention. Histone deacetylase inhibitors (HDACi) increase the acetylation of lysine residues in nucleosomal histones. This reduces their affinity for DNA and leads to transcriptionally active chromatin and the expression of multiple stress response genes.^5^ Evidence of efficacy in preclinical models of organ injury and has led us to hypothesize that HDACi may have clinical utility as organ protection interventions. The aim of the current study was to systematically review the evidence from these experimental studies and to evaluate differences in the effects of different HDACi and modes of administration across a range of experimental models with a view to the design of early phase clinical trials.

## METHODS

Search methods, data extraction, assessment, and presentation were performed as recommended by the Cochrane Handbook for Systematic Reviews of Interventions (Version 5.1).^6^

### Information sources

Potentially eligible studies were identified by searching NCBI, SCOPUS and Ovid database from inception until April 2018 with the following search terms: [(in vitro OR tissue OR cells OR ex vivo OR animal OR human) AND (ischemia reperfusion OR ischemia OR glucose deprivation OR ischemia OR hypoxia OR shock OR trauma OR infarct) AND (brain OR heart OR kidney OR liver) AND (valproate OR HDAC OR epigenetic OR histone acetylation)].

### Search quality

To assess the search quality, all the searches were done in duplicate by S.Y. with default settings from 1960 up to April 2018. Twenty five percent of the titles were randomly selected and cross referenced between searched lists.

### Study selection

**Fig 1:**
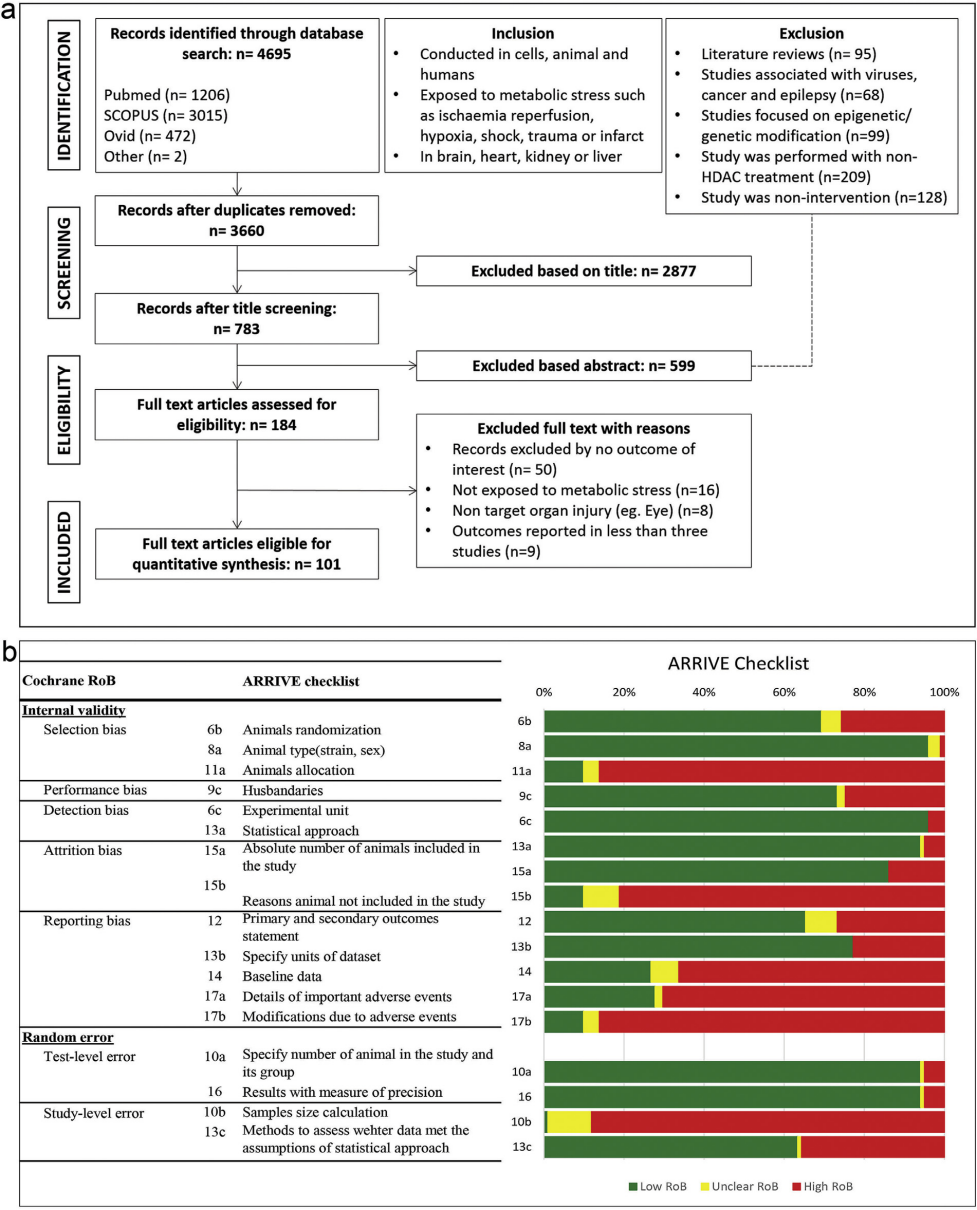
PRISMA flow diagram and methodological quality assessment. (a) PRISMA flow diagram for the systematic review detailing the database searches, numbers of abstract screened, full text assessment with its inclusion and exclusion criteria, and the full text article included for quantitative synthesis. (b) Risk of bias summary: Review author's judgment in 101 included studies based on ARRIVE (Animal Research: Reporting of In Vivo Experiments) checklist.

Two reviewers (S.Y., M.R.) independently selected eligible studies according to the prespecified inclusion and exclusion criteria. All disagreements were resolved by discussion. Following exclusion of titles that were clearly outside the scope of the review, abstracts of the remaining studies were assessed and excluded if they met any of the following criteria: (1) study was a review paper, (2) study was related to cancer/epilepsy/disease, (3) study was undertaken solely on epigenetic/genetic modification, (4) study was performed with non-HDAC treatment, or (5) study was a nonintervention. The full articles for the remaining papers were retrieved and subjected to full text assessment. The inclusion criteria were: (1) Study was conducted in animals, humans and cells, (2) Experimental model of acute organ injury such as ischemia reperfusion, hypoxia, shock, trauma or infarction, or (3) Study was performed in brain, heart, kidney or liver. Studies were further excluded if: (1) they did not assess one of our predefined outcomes listed in the section below, or (2) did not evaluate our prespecified target organs of interest (e.g., eyes), (3) outcomes reported in less than 3 studies (Fig 1).

### Types of outcomes measures

The primary outcome was mortality (dichotomous). Secondary outcomes included a total of 45 variables assessing organ injury (continuous) that were identified in scoping searches and grouped into 8 prespecified outcome categories; **Category 1: Heart injury** (8 variables) included cardiac output, heart infarct size, heart diastolic pressure (dp), heart dP/dT, heart end diastolic pressure (EDP), heart rate, mean arterial pressure (MAP), and rate pressure product (RPP). **Category 2: Brain injury** (6 variables) included Infarct size, lesion volume, neuroscore, time on rotarod, glial fibrillary acidic protein (GFAP), and brain-derived neurotrophic factor (BDNF). **Category 3: Kidney injury** (2 variables) included serum creatinine (Cr) and blood urea nitrogen (BUN). **Category 4: Inflammation** (5 variables) included interleukin-10 (IL-10), interleukin-8 (IL-8), interleukin-6 (IL-6), tumor necrosis factor alpha (TNFa), and cyclooxygenase-2 (COX-2). **Category 5: Cell survival signaling** (12 variables) including nuclear factor kappa B (NF-kB), thiobarbituric acid reactive substances (TBARS), alpha smooth muscles actin (α-sma), beta catenin (β-catenin), heat shock protein 70 (HSP70), inducible nitric oxide synthase (iNOS), matrix mellatoproteinases (MMP-2), myeloperoxidase (MPO), phosphorylated extracellular receptor kinase (pERK), glycogen synthase Kinase 3 β (GSK3β). **Category 6: Measures of homeostasis** (3 variables) included glucose, hemoglobin, and lactate levels. **Category 7: Markers of programmed cell death** (PCD) (7 variables) included apoptosis, terminal deoxynucleotidyl transferase dUTP nick end labeling (TUNEL), apoptotic activator (BAX), B-cell lymphoma 2 (Bcl-2) and caspase-3 (Cas-3), and p53. **Category 8:** Liver injury (2 variables) included alanine aminotransferase (ALT) and AST.

### Data extraction

Data extraction was performed by 2 independent authors (S.Y., B.E.) using a standardized proforma as follows: author, journal, year of publication, animal species, strain, gender, weight, drug administration time, type of injury, type of HDACi, class of HDACi, and concentrations of HDACi. For HDACi classes, these were categorized into: Class I, Class II, Class I/II, and Class III. For type of HDACi; valproic acid (VPA), trichostatin A (TSA), sodium butyrate (SB), and other HDACi-related drugs were extracted. The experimental organ injury was classified as ischemia, trauma, and infection. For each comparison, the number of animals in each group, as well as the mean and standard deviation (SD) or standard error (SEM) for continuous outcomes and the number of events for dichotomous outcomes were extracted. Where the outcomes were reported graphically but not as numerical data in the text, the software WebPlot Digitizer- Version 4.1 (https://automeris.io/WebPlotDigitizer)^7^ was used to extract the values from the graphs. If a published paper involved multiple groups (e.g., using different inhibitors or different concentration), data from each group was individually extracted. Where there were multiple comparisons from the same paper, the data were treated in pair-wise manner and included in the analysis separately. This included multiple independent comparisons reported in the same paper, or multiple treatment comparisons against the same control group. For outcomes measured over time from the same group of animals, we used the first measured time point for analysis. For studies measuring the same outcome in blood and organ tissue from the same animals, we analyzed the measurements taken from blood. Data consistency was cross checked between two independent extraction files and if any inconsistency occurred, the data were cross checked and agreement reached by consensus.

### Assessment of methodological quality

Methodological quality was assessed by two reviewers (S.Y., M.R.) against the ARRIVE checklist.^8^ A random sample of papers were cross checked and disagreements were resolved by consensus. Methodological quality was expressed using graphics adapted from the Cochrane Handbook of Systematic Reviews Collaboration.^9^ Papers were judged to be at low risk of bias if this was evident in all the ARRIVE checklist items.

### Data synthesis

Treatment effects were expressed as the risk ratio (RR) for dichotomous outcomes, and as standardized mean difference (SMD) for continuous outcomes, for HDACi values versus Controls. Multivariate meta-analytic models were used to account for nonindependence in observed effects. To account for repeated use of the same control group in multiple-armed studies, we estimated the variance-covariance matrix of the effect sizes based on Glesser 2009,^10^ and fitted a multivariate random-effects model. In addition, the model included a multilevel structure that takes into account multiple independent comparisons nested within the same papers. All analyses were conducted using the R-package “*metaphor”.* The results were presented using table and forest plots. For the primary analysis, we grouped mouse and rats as rodent.

### Assessment of heterogeneity and reporting biases

Heterogeneity was assessed by using the Q-statistics and its p value, which tests whether the variability in the effect sizes is larger than one would expect based on sampling variability alone. We investigated heterogeneity by performing subgroup analyses. We conducted moderator tests followed by subgroup analyses if moderators were identified. Prespecified subgroups included: animal type (rats, mice), inhibitor class (Class I, II, III, I/II), inhibitor (VPA, TSA, SB, other), type of injury (ischemia, sepsis, trauma or other), and first drug administration time (0–6 hours, 6–24 hours, >24 hours for both pre- and postinhibitor administration). If there were 10 or more papers in the meta-analysis publication bias were investigated by using funnel plots and Egger's test.

## RESULTS

### Searches

A total of 4695 records were retrieved through electronic searches from: PubMed (n = 1206), SCOPUS (n = 3015), OviD (n = 472), and cross reference sources (n = 2). After the exclusion of duplicates (n = 1035), titles clearly outside the scope of the review were excluded (n = 2877). Following the review of titles and abstracts, 599 studies were excluded because they were reviewed manuscripts (n = 95), associated with viruses, cancer and epilepsy (n = 68), focused on genetic/epigenetic modification (n = 99), were performed with non-HDACi treatment (n = 209), or were noninterventional studies (n = 128). A total of 184 manuscripts underwent detailed review; 50 did not report our prespecified outcome measures, 8 studied nontarget organs (e.g., eye), 16 study did not evaluate prespecified metabolic stress, and 9 study reporting outcomes for less than 3 comparisons. In total 101 manuscripts were included in the quantitative and qualitative analysis. (Fig 1**a**).

### Included studies

The characteristics of included studies are summarized in Table I**,** and **Supplemental Table S1**. The 101 manuscripts identified in searches reported a total of 448 comparisons due to the evaluation of multiple independent interventions within single studies. The experimental models included rodents (n = 92, 414 comparisons) and swine (n = 9, 34 comparisons).

**Table I:** Summary of included studies characteristics and outcomes measured in this systematic review

		Rodent		Swine		Total paper	Total comparison
		Paper	Comparison	Paper	Comparison		
**Animal**	**Injury**						
Mice	Ischemia	24	109			24	109
	Sepsis	6	31			6	31
	Trauma	4	13			4	13
**Mice total**		**34**	**153**			**34**	**153**
Pig	Ischemia			3	10	3	10
	Trauma			6	24	6	24
**Pig total**				**9**	**34**	**9**	**34**
Rat	Ischemia	45	206			45	206
	Sepsis	1	7			1	7
	Trauma	12	48			12	48
**Rat total**		**58**	**261**			**58**	**261**
**Grand total**		**92**	**414**	**9**	**34**	**101**	**448**
**Inhibitor class**	**Inhibitor type**						
I	MGCD0103	1	3			1	3
	Mocetinostat	2	11			2	11
	MS-275	3	4			3	4
	PD-106	1	1			1	1
	SB	9	33			9	33
	Scriptaid	1	3			1	3
**I Total**		**17**	**55**			**17**	**55**
I_II	4-PBA	1	2			1	2
	AN-7	1	8			1	8
	ITF2357	1	6			1	6
	LB-205	1	2			1	2
	PBA	2	14			2	14
	SAHA	10	29			10	29
	TSA	19	82			19	82
	VPA	41	169	9	34	50	203
**I_II Total**		**70**	**312**	**9**	**34**	**79**	**346**
II	MC1568	1	1			1	1
	TubA	6	27			6	27
**II Total**		**7**	**28**			**7**	**28**
III	RGFP966	1	4			1	4
	SAB	2	7			2	7
	Sirtinol	1	6			1	6
**III Total**		**4**	**17**			**4**	**17**
**Grand total**		**91**	**412**	**9**	**34**	**100**	**446**
**Category**	**Outcomes**						
Brain injury	BDNF	4	4			4	4
	Brain infarct	19	36			19	36
	GFAP	4	4			4	4
	Lesion volume	7	10	4	4	11	14
	Neurological score	15	22			15	22
	Rotarod	7	10			7	10
**Brain injury total**		**35**	**86**	**4**	**4**	**39**	**90**
Cell survival signaling	AKT	5	5			5	5
	b-catenin	3	5			3	5
	GSH	5	7			5	7
	HSP70	13	14			13	14
	iNOS	5	5			5	5
	MMP-2	5	7			5	7
	MPO	8	8			8	8
	NFkB	4	5			4	5
	pAkt	8	9			8	9
	P-ERK	4	6			4	6
	TBARS	5	6			5	6
**Cell survival signaling total**		**40**	**77**			**40**	**77**
Heart injury	Cardiac output		3	3	3	3	
	Heart dp	6	7			6	7
	Heart dp_dt	10	14			10	14
	Heart edp	8	9			8	9
	Heart infarct	7	11			7	11
	Heart rate	10	12	5	5	15	17
	MAP	7	11	7	7	14	18
	RPP	7	9			7	9
**Heart injury total**		**21**	**73**	**8**	**15**	**29**	**88**
Inflammation	COX-2	5	5			5	5
	IL-10	3	7			3	7
	IL-1b	9	11			9	11
	IL-6	11	13			11	13
	TNFa	17	23			17	23
**Inflammation total**		**24**	**59**			**24**	**59**
Kidney injury	BUN	7	8			7	8
	Creatinine	7	9			7	9
**Kidney injury total**		**10**	**17**			**10**	**17**
Liver injury	ALT	5	6			5	6
	AST	6	7			6	7
**Liver injury total**		**6**	**13**			**6**	**13**
Markers of PCD	Apoptosis	6	6			6	6
	BAX	4	4			4	4
	Bcl-2	10	13			10	13
	BrdU	4	5			4	5
	Caspase-3	16	18			16	18
	p53	5	6			5	6
	TUNEL	7	8			7	8
**Markers of PCD total**		**32**	**60**			**32**	**60**
Measures of homeostasis	Glucose	5	9			5	9
	Hb	5	9	7	7	12	16
	Lactate	7	11	8	8	15	19
**Measures of homeostasis total**		**11**	**29**	**8**	**15**	**19**	**44**
**Grand total**		**92**	**414**	**9**	**34**	**101**	**448**

*Abbreviations:* α-sma, α smooth muscles actin; ALT, alanine aminotransferase; AST, aspartate aminotransferase; BAX, apoptotic activator; Bcl-2, B-cell lymphoma 2; BDNF, brain-derived neutrophic factor; COX-2, cycloocygenase-2; dp, diastolic pressure; edp, end diastolic pressure; GFAP, glial fibrillary acidic protein; HSP70, heat shock protein 70; IL-6, interleukin 6; IL-1β, interleukin 1β; IL-10, interleukin 10; iNOS, inducible nitric oxide synthase; MAP, mean arterial pressure; MMP-2, matrix mellatoproteinases 2; MPO, myeloperoxidase; NFkB, nuclear factor kappa B; PCD, programmed cell death; pERK, phosphorylated extracellular receptor kinase; RPP, rate pressure product; TBARS, thiobarbituric acid reactive substances; TNFα, tumor necrosis factor α; TUNEL, terminal deoxynucleotidyl transferase dUTP nick end labeling.

The most common experimental injury was ischemia (n = 72, 325 comparisons), followed by trauma (n = 22, 85 comparisons), and sepsis (n = 7, 38 comparisons). More than one type of HDACi was evaluated in some studies. The classes of inhibitors used were: Class I (17 studies, 55 comparisons), Class II (7 studies, 28 comparisons), Class I/II (80 studies, 348 comparisons), and Class III (4 studies, 17 comparisons) including 21 different inhibitors. The most commonly used inhibitors were: Valproic acid (VPA) in 50 studies, 203 comparisons, trichostatin A (TSA) in 19 studies with 82 comparisons, sodium butyrate (SB) in 9 studies with 33 comparisons, suberoylanilide hydroxamic acid (SAHA, Vironostat) in 10 studies with 29 comparisons and tubastatin A (TubA) in 6 studies with 27 comparisons.

All study characteristics and findings are listed in **Supplemental Tables S1** and summarized in Tables I and 2.

**Table II:** Primary analysis output

*Primary analysis*					
Variable	Paper	Comparisons	SMD (95% CI)	p value	QE (df, p value)
**Brain injury**					
BDNF	4	4	2.38 (0.88–3.88)	0.0018	QE = 8.83 (df = 3, p = 0.032)
Brain infarct	19	36	−1.70 (−2.22 to −1.18)	<0.0001	QE = 156.01 (df = 35, p < 0.0001)
GFAP	4	4	−1.93 (−2.81 to −1.05)	<0.0001	QE = 1.63 (df = 3, p = 0.653)
Lesion volume	7	10	−1.13 (−1.81 to −0.45)	0.0011	QE = 31.19 (df = 9, p = 0.000)
Rotarod	7	10	1.15 (0.25–2.06)	0.0126	QE = 32.92 (df = 9, p = 0.000)
**Inflammation**					
IL-10	3	7	3.84 (0.34–7.35)	0.0316	QE = 88.51 (df = 6, p < 0.0001)
IL-1b	9	11	−2.31 (−3.62 to −1.01)	0.0005	QE = 67.80 (df = 10, p < 0.0001)
IL-6	11	13	−1.68 (−2.80 to −0.56)	0.0033	QE = 173.17 (df = 12, p < 0.0001)
TNFa	17	23	−1.59 (−2.68 to −0.50)	0.0042	QE = 246.39 (df = 22, p < 0.0001)
**Heart injury**					
Heart dp	6	7	1.90 (1.25–2.55)	<0.0001	QE = 10.00 (df = 6, p = 0.125)
Heart dp_dt	10	14	1.50 (0.78–2.22)	<0.0001	QE = 55.14 (df = 13, p < 0.0001)
Heart edp	8	9	−1.32 (−2.56 to −0.09)	0.0354	QE = 54.41 (df = 8, p < 0.0001)
Heart infarct	7	11	−2.34 (−3.82 to −0.86)	0.0019	QE = 58.46 (df = 10, p<0.0001)
**Kidney injury**					
RPP	7	9	1.27 (0.58–1.96)	0.0003	QE = 21.58 (df = 8, p = 0.006)
BUN	7	8	−0.82 (−1.31 to −0.33)	0.0010	QE = 19.06 (df = 7, p = 0.008)
**Markers of PCD**					
BAX	4	4	−3.46 (−6.82 to −0.09)	0.0440	QE = 42.92 (df = 3, p<0.0001)
Bcl-2	10	13	4.08 (1.94–6.21)	0.0002	QE = 76.16 (df = 12, p<0.0001)
BrdU	4	5	4.10 (2.35–5.84)	<0.0001	QE = 8.79 (df = 4, p = 0.066)
Caspase-3	16	18	−1.74 (−3.42 to −0.06)	0.0424	QE = 318.71 (df = 17, p<0.0001)
TUNEL	7	8	−4.46 (−6.78 to −2.14)	0.0002	QE = 44.10 (df = 7, p<0.0001)
**Cell survival signaling**					
b-catenin	3	5	1.83 (0.66–3.00)	0.0022	QE = 8.65 (df = 4, p = 0.071)
HSP70	13	14	2.56 (1.87–3.24)	<0.0001	QE = 42.00 (df = 13, p<0.0001)
MPO	8	8	−6.95 (−13.55 to −0.34)	0.0392	QE = 96.59 (df = 7, p<0.0001)
**Brain injury**					
Lesion volume	4	4	−1.52 (−2.39 to −0.66)	0.0006	QE = 5.04 (df = 3, p = 0.169)
**Measures of homeostasis**					
Lactate	8	8	0.80 (0.09–1.51)	0.0270	QE = 19.35 (df = 7, p = 0.007)
**Variable**	**Paper**	**Comparisons**	**RR (95% CI)**	**p value**	**QE (df, p value)**
Survival	15	16	0.53 (0.39–0.71)	<0.0001	QE = 24.40 (df = 15, p = 0.059)
Survival	3	3	0.48 (0.25–0.91)	0.0242	QE = 2.16 (df = 2, p = 0.340)

*Moderator analysis*					
Variable	Animal	Drug class	Inhibitor	Insult	Admin Time
**Brain injury**					
BDNF					
Brain infarct	0.0461				
Lesion volume		0.0050		0.0000	0.0001
Rotarod					
**Inflammation**					
IL-10					
IL-1b	0.0456			0.0205	
IL-6	0.0300				
TNFa					
**Heart injury**					
Heart dp_dt		0.0079			
Heart edp					
RPP			0.0006		
Heart infarct					0.0088
**Kidney injury**					
BUN		0.0196			
**Markers of PCD**					
BAX		0.0015			
Bcl-2			0.0011		
Caspase-3		0.0010			
TUNEL					
**Cell survival signaling**					
HSP70	0.0283		0.0076		
MPO					
**Measures of homeostasis**					
Lactate					

Abbreviations: BAX, apoptotic activator; Bcl-2, B-cell lymphoma 2; BDNF, brain-derived neutrophic factor; COX-2, cycloocygenase-2; dp, diastolic pressure; edp, end diastolic pressure; GFAP, glial fibrillary acidic protein; HSP70, heat shock protein 70; IL-6, interleukin 6; IL-1β, interleukin 1β; IL-10, interleukin 10; MPO, myeloperoxidase; PCD, programmed cell death; RPP, rate pressure product; TNFα, tumor necrosis factor α; TUNEL, terminal deoxynucleotidyl transferase dUTP nick end labeling.

Data presented as treatment effect as risk ratios (RR) for survival and standardized mean difference (SMD) for dichotomous outcomes and its p-value. Secondary analysis of moderator effect with Q-statistics and its p value.

The primary outcome of experimental mortality was reported in 16 rodent studies and 3 swine studies.

Brain injury was assessed in rodents and swine through the following variables: BDNF, brain infarct, GFAP, lesion volume, neurological score and rotarod and were reported in total 4, 19, 4, 7, 15, and 7 studies, respectively.

Heart injury was evaluated in rodents and swine through cardiac output, heart dP, heart dP/dT ratio, heart EDP, size of heart infarct, heart rate, MAP, and RPP. These variables were reported in 3, 6, 10, 8, 7, 15, 14, and 7 studies, respectively.

Kidney injury in rodents was assessed through the BUN and creatinine in 14 analyses studies, while liver injury was assessed by measuring the ALT and AST levels in a total of 11 studies. Inflammation markers selected in rodent studies were: COX-2, IL-10, IL-1β, IL-6, and TNF-α, and reported in 24 studies. Measures of homeostasis included glucose, hemoglobin, and lactate levels and were reported in19 studies. Cell survival signaling was evaluated by measuring: α-SMA, AKT, β-catenin, GSH, HSP70, iNOS, MMP-2, MPO, NFkB, P-ERK, pAkt, and TBARS reported in 40 rodent studies.

Markers of programmed cell death (PCD) assessment comprised of: apoptosis, BAX, BCL-2, BrDU, Caspase-3, p53, and TUNEL. These were reported in 32 rodent studies.

### Assessment of methodological quality

The grouped assessment of methodological quality as measured against the ARRIVE checklist is reported in Fig 1b. Assessment of methodological quality for individual studies is reported in Supplemental Table S2. No study was free from important methodological limitations: 87/101 study did not specify the animal allocation, 82/101 studies did not describe the reasons animals included in the study were excluded from the analyses, 67/101 studies does not provide baseline data of the studies, 71/101 papers did not report the adverse events attributable to the intervention, and 87/101 did not specify any modifications made due to adverse events. Finally, 89/101 studies did not include the sample size calculation in their experimental design.^11^, ^12^, ^13^, ^14^, ^15^, ^16^, ^17^, ^18^, ^19^, ^20^, ^21^, ^22^, ^23^, ^24^, ^25^, ^26^, ^27^, ^28^, ^29^, ^30^, ^31^, ^32^, ^33^, ^34^, ^35^, ^36^, ^37^, ^38^, ^39^, ^40^, ^41^, ^42^, ^43^, ^44^, ^45^, ^46^, ^47^, ^48^, ^49^, ^50^, ^51^, ^52^, ^53^, ^54^, ^55^, ^56^, ^57^, ^58^, ^59^, ^60^, ^61^, ^62^, ^63^, ^64^, ^65^, ^66^, ^67^, ^68^, ^69^, ^70^, ^71^, ^72^, ^73^, ^74^, ^75^, ^76^, ^77^, ^78^, ^79^, ^80^, ^81^, ^82^, ^83^, ^84^, ^85^, ^86^, ^87^, ^88^, ^89^, ^90^, ^91^, ^92^, ^93^, ^94^, ^95^, ^96^, ^97^, ^98^, ^99^, ^100^, ^101^, ^102^, ^103^, ^104^, ^105^, ^106^, ^107^, ^108^, ^109^, ^110^, ^111^ In summary, no study identified in the review was free from potential bias.^1^

### Primary outcome

**Fig 2:**
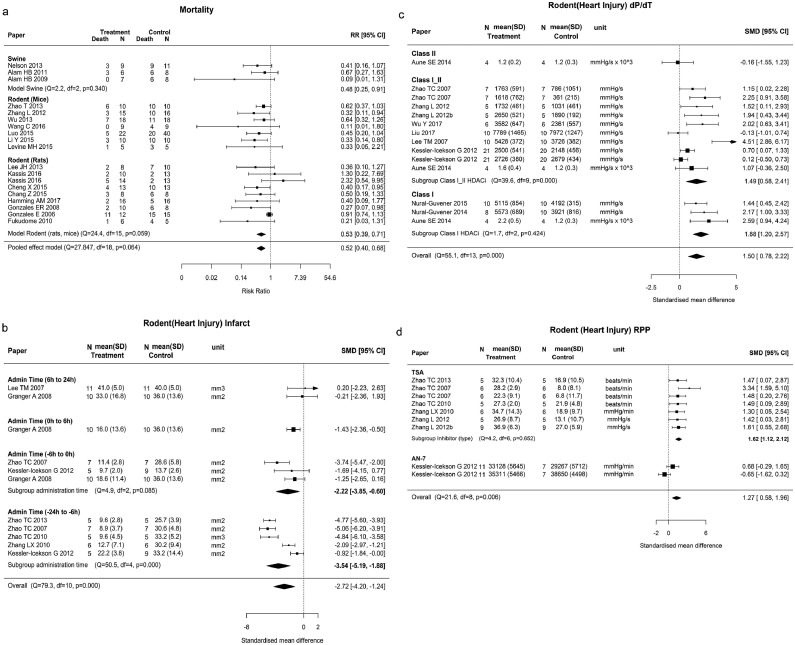
Forest plots for primary outcome (mortality) and secondary outcomes of heart injuries. (a) Mortality for rodent and swine. (b) Rodent heart infarct by first administration time. (c) Rodent heart dP/dT by inhibitor class, (d) Rodent heart RPP by types of inhibitor. Effect size was presented as SMD (95% CI) and heterogeneity test was presented as (Q statistics, df, p value). N, number of animals; SMD, standardized mean difference; SD, standard deviation; CI, confidence interval; df, degree of freedom; RPP, rate pressure product.

Pooled effect estimates for mortality favored the use of HDACi in both rodents, RR 0.53 (95% confidence interval [CI] 0.4–0.7, p < 0.0001, Q = 24.40, p = 0.059) and swine RR 0.48 (95% CI 0.25–0.91, p = 0.024) (Q = 2.16, p = 0.340), (Fig. 2a). The pooled risk ratio for mortality from all studies was RR = 0.52, (95% CI 0.40–0.68 p < 0.001) without heterogeneity (Q = 27.85, p = 0.064).

### Secondary outcomes

**Brain injury**: The pooled effect estimate favored HDACi treatment over controls for the outcomes brain infarct size (SMD −1.70, 95% CI −2.22 to −1.18, p < 0.0001, 19 studies), brain lesion volume (SMD −1.13, 95% CI −1.81 to −0.45, p = 0.001, 7 studies), time on rotarod (SMD 1.15, 95% CI 0.25–2.06, p = 0.013, 7 studies), BDNF levels (SMD 2.38, 95% CI 0.88–3.88, p = 0.002, 4 studies), and glial fibrillary acidic protein (GFAP) (SMD −1.93, 95% CI −2.81 to −1.05, p < 0.0001, 4 comparisons) when compared with untreated animals.

Heterogeneity was significant for all outcomes except GFAP (Q =  2.39, p = 0.653). In the swine studies, HDACi resulted in significantly lower brain lesion volumes (SMD −1.52, 95% CI −2.39 to −0.66, p = 0.001) without heterogeneity (Q= 5.04, p = 0.169) (Table II).

**Heart injury**: The pooled effect estimate favored HDACi treatment over controls for infarct size (SMD −2.34, 95% CI −3.82 to −0.86, p < 0.001, 7 studies), EDP (SMD −1.32, 95% CI −2.56 to −0.09, p = 0.03, 8 studies), RPP (SMD 1.27, 95% CI 0.58−1.96, p < 0.0001, 7 studies), dP/dT ratio (SMD 1.50, 95% CI 0.78–2.22, p < 0.0001, 10 studies), and heart dP (SMD 1.90, 95% CI 1.25–2.55, p < 0.0001, 6 studies). Heterogeneity was not significant for heart dP (Q = 10, p = 0.125). There was heterogeneity for dP/dT (Q = 55.14, p < 0.0001), infarct size (Q = 58.46, p < 0.0001), and RPP (Q = 21.58, p < 0.05) (Table II).

**Kidney injury** was reduced by HDACi as determined by serial BUN (SMD −1.06, 95% CI −1.41 to −0.70, p < 0.001, 12 studies) with significant heterogeneity (Q = 25.4, p = 0.021) (Table II).

Liver injury: Liver AST and ALT were not significantly different between treatment and control groups **Supplemental Table S3.**

Inflammation: The pooled effect estimate favored HDACi treatment over controls for IL-1β (SMD −2.13, 95% CI −3.62 to −1.01, p = 0.001, 9 studies), IL-6 (SMD −1.68, 95% CI −2.80 to −0.56, p = 0.003, 11 studies), and TNF-α (SMD −1.59, 95% CI −2.68 to −0.50, p = 0.004, 17 studies) were lower and IL-10 was higher (SMD 3.84, 95% CI 0.34−7.35, p = 0.032, 3 studies). There was significant heterogeneity for all analyses (Table II).

**Programmed cell death (PCD)** BAX, Caspase 3, and TUNEL were lower in the HDACis treatment groups, while Bcl-2 and BrdU were higher. Heterogeneity was significant for all outcomes with the exception of BrdU (Q = 8.80, p = 0.066) (Table II).

**Cell survival signaling** The pooled effect estimate favored HDACi treatment over controls for β-catenin (SMD 1.83, 95% CI 0.66 to 3.00, p = 0.002, 3 studies) and HSP70 (SMD 2.56, 95% CI 1.87–3.24, p < 0.001, 13 studies) and MPO (SMD −6.95, 95% CI −13.55 to −0.34, p = 0.039, 8 studies). There was heterogeneity for all analyses except β-catenin (Q = 8.65, p = 0.070) (Table II).

**Publication bias** Funnel plots for all primary and secondary outcomes are shown in **Supplemental Figure S1.** Where results on more than 10 studies were reported there was evidence of significant reporting bias (Egger's test, p < 0.05) for brain infarct size, heart dP/dT, kidney BUN, kidney Creatinine, IL-1B, IL-6, TNFa, Bcl-2, Caspase-3 and HSP-70.

### Subgroup analyses

To investigate sources of heterogeneity (rodents 19 primary analyses, swine 0 primary analyses) we conducted moderator analyses to examine characteristics of the HDACi treatment and/or type of injuries associated with the overall effect estimate. If moderators were identified heterogeneity was further explored using subgroup analyses (Table II).

### Rodent studies

In myocardial protection the effect of the moderator timing of HDACi administration relative to the time of injury was significant for Heart Infarct size (p = 0.009). The effect in heart protection was greater when HDACi were administered before versus after the injury. HDACi administration 6–24 hours before the injury (SMD −3.54, 95% CI −5.19 to −1.88) had a greater effect than administration within 6 hours of the injury (SMD −2.22, 95% CI −3.85 to −0.60). (Fig 2, **b**). For heart dP/dT ratio and RPP in rodents effect sizes were moderated by the inhibitor class or type of inhibitors (Fig 2, **c and d**). For Heart dP/dT, the effect size for class I HDACis on dP/dT (SMD 1.88, 95% CI 1.20–2.57, p = 0.424) was significantly higher than that for class I/II (SMD 1.49, 95% CI 0.58–2.41) with the moderator test for subgroup differences p = 0.008. The administration of TSA-induced higher heart RPP than the controls (SMD 1.62, 95% CI 1.12–2.12) with little heterogeneity (Q = 4.2, p = 0.652) with moderator test for subgroup differences p = 0.006.

**Fig 3:**
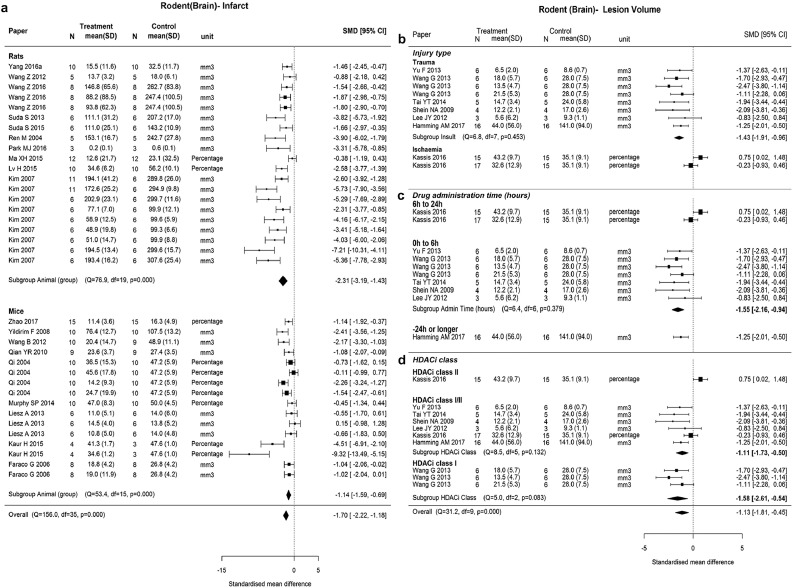
Forest plots for brain injury outcomes. (a) Rodent brain infarct by animal types. (b–d) Rodent brain lesion volume by injury type, first administration time and HDACi class. Effect size was presented as SMD (95% CI) and heterogeneity test was presented as Q statistics, df, and p value. N, number of animals; SMD, standardized mean difference; SD, standard deviation; CI, confidence interval; df, degree of freedom; HDACi, histone deacetylase inhibitor.

In brain protection, effect sizes were moderated by animal type (p = 0.046). The reduction in brain infarct size was larger in rats (SMD −2.31, 95% CI −3.19 to −1.43) compared to mice (SMD −1.14, 95% CI −1.59 to −0.69), although heterogeneity within the rat and mouse subgroups remained high (p < 0.001) (Fig 3, **a**). For brain lesion volume post trauma potential moderators included injury type (p < 0.001), administration time (p < 0.001), and inhibitor class (p = 0.005). Treatment by HDACi showed a significant reduction in brain lesion volume for induced trauma (SMD −1.43, 95%CI −1.91 to −0.96) with little heterogeneity (Q = 6.8, p = 0.453) (Fig 3, **b**). There was also significant reduction in brain lesion volume for administering inhibitors within 6 hours postinjury (SMD −1.55, 95% CI −2.16 to −0.94) with little heterogeneity Q = 6.4, p = 0.379) (Fig 3, **c**). Treatment with HDACi class I showed larger effect size in lesion reduction (SMD −1.58, 95% CI −2.61 to −0.54) compared to HDACi class I/II (SMD −1.11, 95% CI −1.73 to −0.50) (Fig 3, **d**).

**Fig 4:**
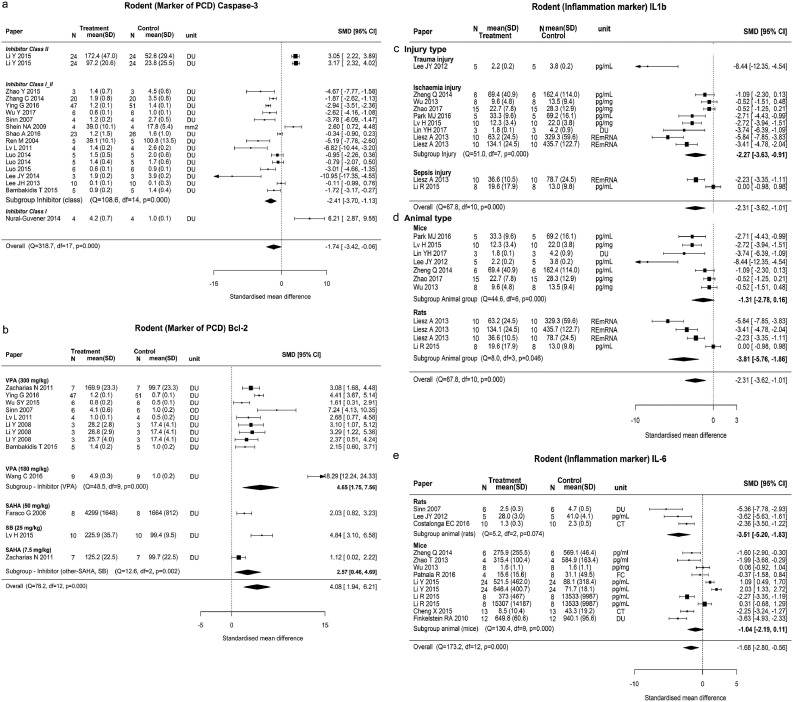
Forest plots for programmed cell death (PCD) markers and inflammation markers. (a) Rodent Caspase-3 by inhibitor class. (b) Rodent Bcl-2 by type of inhibitor. (c, d) Rodent interleukin 1b (IL-1b) by injury type and animal type. (e) Rodent interleukin 6 (IL-6) by animal type. Effect size was presented as SMD (95% CI) and heterogeneity test was presented as Q statistics, df, and p value. N, number of animals; SMD, standardized mean difference; SD, standard deviation; CI, confidence interval; df, degree of freedom; DU, densitometry unit; FC, fold change; CT, cycle threshold.

For markers of programmed cell death, inhibitor class (p = 0.001) and type (p = 0.0011) were moderators for Caspase-3 and BCL-2, respectively. Compared with the controls, the administration of class I/II inhibitors showed significant reduction in Caspase-3 (SMD −2.41, 95% CI −3.70 to −1.13), but the administration of either class I or class II specific inhibitors increased Caspase-3, although these sub-group analyses included <3 published studies (Fig 4, **a**). Bcl-2 was significantly increased by VPA (SMD 4.65, 95% CI 1.75–7.56), but not by other inhibitors (SMD 2.57, 95% CI 0.46, 4.69). Heterogeneity remained high within individual inhibitor groups (p < 0.001) (Fig 4, **b**).

Levels of IL-1β were moderated by the type of injury (p = 0.021), animal (p = 0.046), and IL-6 were moderated by animal (p = 0.030) (Table II). There was significant reduction by HDACi of IL-1β following ischemia (SMD −2.27, 95% CI −3.63 to −0.91) and trauma (SMD −8.44, 95% CI −12.35 to −4.54) but not with other injury types (Fig 4, **c and d**). Reduction in IL-6 was significant in rats (SMD −3.51, 95% CI −5.2 to −1.83) but not in mice (Fig 4, **e**). None of the prespecified moderating variables were found to significantly interact with brain outcomes BDNF and Rotarod, heart injury assessed by EDP, kidney injury outcomes BUN, and creatinine or for COX-2, IL-10 or TUNEL (Table II).

### Sensitivity analyses

No sensitivity analysis stratified by methodological quality was performed as all of the studies were considered at high risk of bias.

## DISCUSSION

### Main findings

HDACi reduce mortality as well as myocardial, brain and kidney injury in experimental models of organ injury. This effect was observed across multiple species and against diverse modes of injury. In models of myocardial injury HDACi reduced myocardial infarct volume whilst increasing measures of myocardial contractility. In models of brain injury HDACi reduced traumatic brain injury and increased functional performance. Organ protection was attributable to increases in pro-survival cell signaling, and reductions in inflammation and programmed cell death. These findings highlight a potential novel application for this class of drugs in clinical settings characterized by acute organ injury.

### Strengths and limitations

This is the first study to our knowledge that has systematically reviewed the experimental evidence for HDACi mediated organ protection. The review used comprehensive search strategies in a wide range of registries and data sources, had access to the full texts of all identified trials, used a contemporary risk of bias assessment, and assessed a wide range of experimental outcomes. The study also had important limitations. First, the quality assessment against the ARRIVE guidelines indicated that all of the 101 included studies had significant methodological limitations and were at risk of bias. Importantly, most studies were lacking data on adverse events which is essential when determining the balance of risks and benefits for any clinical trial. Second, assessment of funnel plots indicated likely publication bias for most outcomes, suggesting that selective reporting may have contributed to our results. This is supported by the observations that no negative published study was identified, and no pre-analysis protocols were reported. Third, heterogeneity was observed for many of the secondary outcomes measures, although analysis of the effects of pre-specified modifiers on heterogeneity indicated that much of the variation was attributable to differences in species, type of injury, and type of drug. In rodent models of myocardial protection the effects of HDACi on infarct size were greatest if the intervention was administered 6–24 hours prior to the intervention, and on myocardial contractility if the intervention was Class I versus Class I/II HDACi, or TSA versus other compounds. These moderators were also significant sources of heterogeneity in models of traumatic brain injury where effects were greater when HDACi were administered within 6 hours of injury. Fourth, we included 4 studies that evaluated class III HDACi (sirtuin inhibitors) that act via mechanisms distinct from Class I, II, and IV HDACi. These studies were identified by our prespecified eligibility criteria and were therefore included in our analyses. A *post–hoc* analysis has demonstrated that their inclusion did not materially alter our results (data not shown).

### Clinical importance

The limitations of the data notwithstanding the results demonstrate that HDACi reduce mortality in experimental models by conferring multi-organ protection often following a single treatment administered in some cases post injury. We speculate that these findings are consistent with a genome wide activation of stress response genes via an epigenetic process or mitochondrial protection signalling.^112^ This was not proven by the current analysis however as the evaluation of the mechanisms of action of HDACi in these studies was limited. Additionally, uncertainty as to the mechanism of action was also evident in an early phase I trial in healthy humans. Here sodium valproate administered as a single dose (120 mg/kg over 1 hour) resulted in changes in leucocyte signaling homologous to those reported in the current analysis, however these changes were not attributed to alterations in histone acetylation.^113^

Other areas of uncertainty relate to the most effective HDACi and the timing of administration. In the current analysis TSA had greater efficacy than VPA however as yet this drug has not been evaluated in clinical trials.^114^ TSA has greater specificity for HDACi relative to VPA, supporting our primary hypothesis, and further evaluation of pan-HDACi is clearly warranted. Of the many HDACi currently undergoing clinical evaluation in cancer, HIV infection and neurological diseases Vorinostat (SAHA) has been shown to be the most promising and with acceptable toxicity.^115^ In this review Vorinostat was evaluated in 11 studies (31 comparisons) where it was shown to be effective. VPA the Class I/II HDACi evaluated most often in preclinical studies is inexpensive and already widely used in neurological disease. However, even short courses of VPA have significant toxicity, particularly in elderly patients.^116^, ^117^ This may not be clinically important in acute settings such as trauma or infarction where a single large dose will be given postinjury but may have possible sequelae if used for planned procedures such as surgery.

## CONCLUSIONS

In experimental studies HDACi administration results in organ protection against diverse injurious stimuli including ischemia, sepsis, and trauma. Major methodological limitations were identified in all of the studies identified, and importantly, adverse effects, and toxicity were not reported in most studies. HDACi are now undergoing clinical evaluation in multiple clinical settings. The evidence presented here supports their early phase evaluation as organ protection interventions.

## Appendix Supplementary materials

Image, application 1

Table S1 **Summary of study characteristics and data extraction included in this systematic review.** Table represents further detailed study characteristics and its collected data for this systematic review as discussed in Table 1 and Table 2. Image, image 2
