# Supplementary figures and images for: Systematic review and meta-analysis of experimental studies evaluating the organ protective effects of histone deacetylase inhibitors
Source: Transl Res. 2019 Mar;205:1–16. doi: 10.1016/j.trsl.2018.11.002 (PMC6386580; doi:10.1016/j.trsl.2018.11.002)

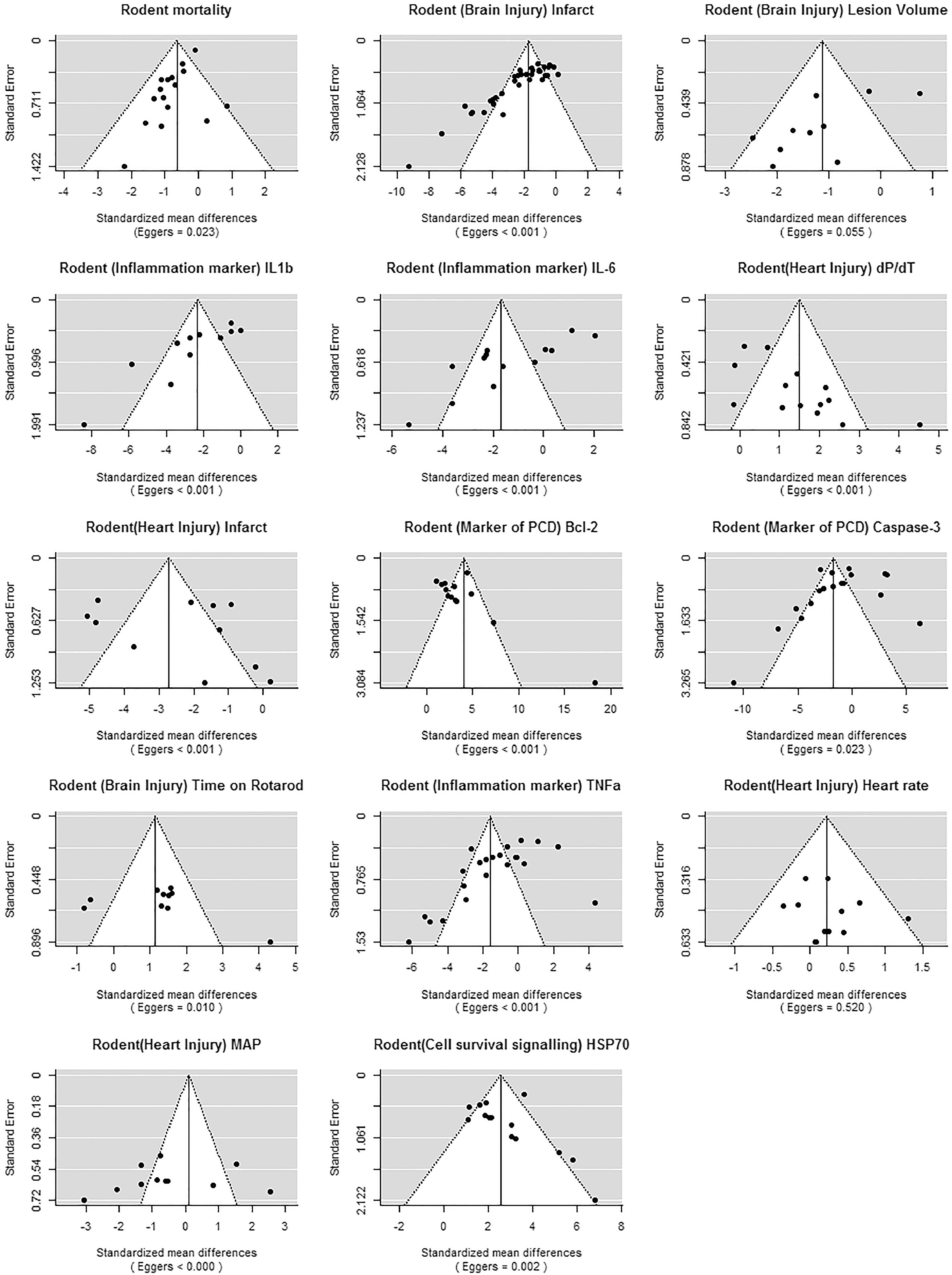

Supplement: Table S1 — Summary of study characteristics and data extraction included in this systematic review. Table represents further detailed study characteristics and its collected data for this systematic review as discussed in Table 1 and Table 2. [file mmc2.jpg]
